# Systemic attenuation of the TGF-β pathway by a single drug simultaneously rejuvenates hippocampal neurogenesis and myogenesis in the same old mammal

**DOI:** 10.18632/oncotarget.3851

**Published:** 2015-05-06

**Authors:** Hanadie Yousef, Michael J. Conboy, Adam Morgenthaler, Christina Schlesinger, Lukasz Bugaj, Preeti Paliwal, Christopher Greer, Irina M. Conboy, David Schaffer

**Affiliations:** ^1^ Department of Bioengineering and California Institute for Quantitative Biosciences (QB3), UC Berkeley, Berkeley, CA, USA; ^2^ Department of Molecular and Cellular Biology, UC Berkeley, Berkeley, CA, USA; ^3^ Department of Chemical and Biomolecular Engineering, UC Berkeley, Berkeley, CA, USA; ^4^ Helen Wills Neuroscience Institute, UC Berkeley, Berkeley, CA, USA; ^5^ Department of Neurology and Neurological Sciences, Stanford University, Stanford, CA, USA

**Keywords:** aging, stem cell microenvironment, neurogenesis, TGF-β signaling, myogenesis

## Abstract

Stem cell function declines with age largely due to the biochemical imbalances in their tissue niches, and this work demonstrates that aging imposes an elevation in transforming growth factor β (TGF-β) signaling in the neurogenic niche of the hippocampus, analogous to the previously demonstrated changes in the myogenic niche of skeletal muscle with age. Exploring the hypothesis that youthful calibration of key signaling pathways may enhance regeneration of multiple old tissues, we found that systemically attenuating TGF-β signaling with a single drug simultaneously enhanced neurogenesis and muscle regeneration in the same old mice, findings further substantiated via genetic perturbations. At the levels of cellular mechanism, our results establish that the age-specific increase in TGF-β1 in the stem cell niches of aged hippocampus involves microglia and that such an increase is pro-inflammatory both in brain and muscle, as assayed by the elevated expression of β2 microglobulin (B2M), a component of MHC class I molecules. These findings suggest that at high levels typical of aged tissues, TGF-β1 promotes inflammation instead of its canonical role in attenuating immune responses. In agreement with this conclusion, inhibition of TGF-β1 signaling normalized B2M to young levels in both studied tissues.

## INTRODUCTION

Adult stem cells persist in the body as we age, but their regenerative capacity declines over time, leading to an inability of tissues and organs to maintain homeostasis and repair damage with advancing age. Evidence suggests that the intrinsic capacity of some tissue stem cells, such as in muscle, for tissue maintenance and repair does not drastically decline with age. Rather, molecular changes in the stem cell microenvironments or niches contribute to or account for diminished stem cell regenerative potential in several tissue types, ultimately leading to tissue aging. Such changes in stem cell microenvironments that negatively regulate stem cell function have been observed in aged skeletal muscle, brain, skin, blood, and bone [1]. Considering that the same morphogenic signal transduction pathways – including Notch, Wnt, transforming growth factor β (TGF-β), fibroblast growth factor (FGF), Sonic hedgehog (Shh), and others – regulate adult stem cell behavior in different tissues [2], it is possible that age-related changes in these pathways that lead to diminished stem cell regeneration are also conserved among multiple organ systems, such as brain and muscle. Furthermore, complex interplays between systemic and local changes in specific signaling factors with age have been shown to inhibit stem cell mediated tissue regeneration [3–9], and these interactions may also be conserved across tissues.

Neurogenesis occurs throughout life in the subgranular zone (SGZ) of the dentate gyrus (DG) of the hippocampus and the subventricular zone (SVZ) of the lateral ventricles in mammals, via differentiation of adult neural stem cells (NSCs) into excitatory granule neurons and inhibitory olfactory bulb interneurons, respectively (Ming & Song, 2005). Hippocampal neurogenesis is believed to modulate new memory formation, while SVZ neurogenesis plays a role in sensory functions [10–12]. Hippocampal neurogenesis, however, severely declines with age in rodents and primates [13–15]. In addition, SGZ neurogenesis is very active in humans and exhibits a steady decline in both NSC number and function with age [15–17], though the significance of the SVZ to human biology and health is unclear as this region does not exhibit active neurogenesis leading to maintenance of olfactory functions in adult humans [18]. Skeletal muscle regeneration also occurs throughout life, though muscle loses its regenerative capacity with age due to the failure of satellite cells (muscle stem cells) to divide and generate fusion competent myoblasts and terminally differentiated myofibers in response to muscle injury or attrition [1]. Consequentially, the replacement of the damaged tissue with new muscle fibers becomes inefficient with age, and instead scarring and inflammation persist [19, 20]. Considering that TGF-β regulates adult stem cell behavior in various tissues [1, 21–24], including in brain and muscle, we hypothesized that age-related elevation in the intensity of this pathway may contribute to diminished stem cell regeneration in the hippocampal niche, which combined with our prior results with the skeletal muscle stem cell niche would demonstrate conservation of signal transduction changes with age.

TGF-β1 is a multi-functional cytokine that becomes elevated with age both in circulation and locally in several tissues, including the muscle and brain [6, 25]. Such changes in the intensity of TGF- β/pSmad2,3 signaling – with aging, pathology, or experimental induction – have been shown to perturb homeostasis of such diverse tissues as muscle, bone and cartilage, the subventricular zone of the brain, vasculature, the hematopoietic/immune system, heart and skin [21–24, 26–34]. TGF-β1 can inhibit cell proliferation by upregulating the expression of several CDK inhibitors, including p21 [27, 35–37], and it may thereby directly inhibit the proliferation of tissue stem and progenitor cells. Furthermore, TGF-β1 was recently described to be required for late-stage neuronal development, maturation or survival [38]. Increased TGF-β1 signaling could therefore also be a mechanism to enhance mature neuronal differentiation or survival in addition to its detrimental effects on de-novo neurogenesis. However, it may also modulate tissue inflammation, as TGF-β1 can be either anti- or pro-inflammatory depending on the levels of this morphogen and the gene expression landscape of the responding tissue [29, 32]. For example, young and healthy levels of TGF-β1 are known to down-regulate the immune response, and accordingly the complete absence of TGF-β1 or its downstream signaling causes multifocal inflammation in young mammals (reviewed in [32]). In contrast, when present at high or pathological levels, TGF-β1 has been shown to promote massive inflammation in a variety of systems [29, 32]. While inflammatory responses, which in general can include induction of MHC class I and/or class II genes and the production of multiple inflammatory cytokines including TGF-β1, are needed for productive regeneration of tissues including skeletal muscle and brain, prolonged and excessive inflammation is known to interfere with tissue maintenance and repair [7, 29, 32].

We demonstrate an elevation of TGF-β1 in the aged circulation, old muscle, and old brain and cross-tissue conservation in elevated TGF-β/pSmad2,3 signal transduction in aged skeletal muscle and neural stem cell microenvironments. An ability of circulating TGF-β1 to cross the blood brain barrier may increase with age, when BBB becomes more permeable [39], but regardless of its endocrine levels, local production of TGF-β1 by microglia and by endothelial cells increases in the hippocampus with aging. Importantly, the age-associated increase in TGF-β1 levels leads to a decline in NSC proliferation and downstream neurogenesis with age. Moreover, TGF-β may exert its pro-aging effects in brain and muscle in part by increasing inflammatory responses, as indicated by the upregulation of β2 microglobulin (B2M), a component of MHC class I molecules. Finally, youthful calibration of TGF-β1/pSmad2,3 pathway by a systemically administered inhibitor of TGF-β receptor kinase activity reduced tissue inflammation in brain and muscle, as indicated by normalized levels of B2M, and robustly rejuvenated hippocampal neurogenesis and skeletal myogenesis in the same old animal.

## RESULTS

### TGF-β1/pSmad signaling increases in aged neural and muscle stem cell niches

**Figure 1:**
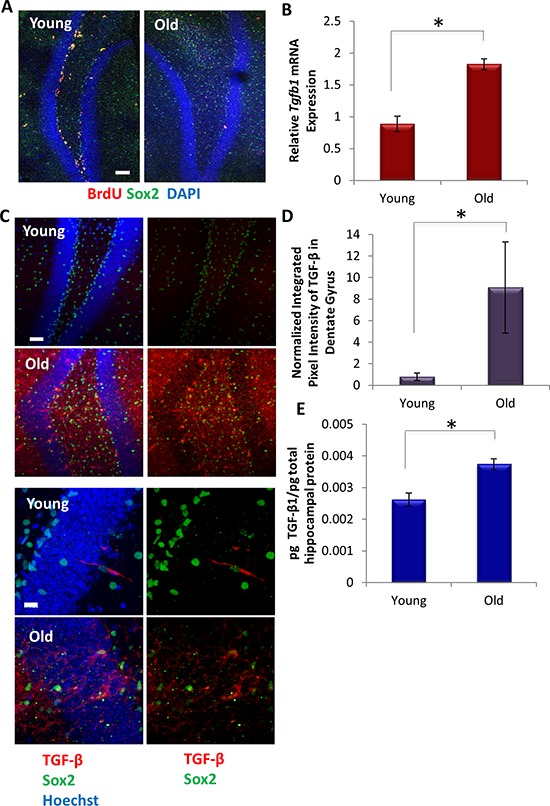
TGF-β increases with age locally in mice hippocampi **A.** Young (2 month) and old (24 month) mice (*n* = 3) were administered daily with BrdU for 5 days, followed by perfusion and PFA fixation. Immunofluorescence (IF) was performed for Sox2 (green) and BrdU (red), with DAPI (blue) labeling all nuclei. Representative images are shown. Scale bar = 100 μM. **B.** qRT-PCR quantification of *Tgfb1* mRNA expression from young and old hippocampi. The relative average expression level was normalized to *GAPDH* and presented as the average expression level relative to that of young hippocampi. Significant differences were identified by Student's *t*-tests (two-tailed) (*p < 0.003). Error bars indicate standard error of the mean (*n* = 4 biological replicates per group). **C.** Immunofluorescence was performed on perfused and PFA fixed young and old brain tissue sections (*n* = 3), for Sox2 (green) and TGF-β1,2,3 (red), with Hoechst (blue) labeling all nuclei. Representative low and high magnification images are shown. Scale bars = 50 μM. **D.** Integrated pixel intensity of TGF-β1,2,3 immunofluorescence in young and old dentate gyri tissue sections was calculated using ImageJ. Pixel intensities are presented relative to young dentate gyri. Significant differences were identified by Student's *t*-tests (*p < 0.003). Error bars indicate standard error of the mean (*n* = 3 young, 3 old biological replicates). **E.** An ELISA was performed on young and old hippocampal protein extract to assess the level of local TGF-β1 and represented as the averages of the pg TGF-β1 normalized to pg total hippocampal protein lysate. Significant differences were identified by Student's *t*-tests (two-tailed) (*p < 0.01). Error bars indicate standard error of the mean (*n* = 4 young, 5 old biological replicates).

Consistent with previously published results [13–15], we found that Sox2+BrdU+ proliferating Type 1 and 2a hippocampal neural stem and progenitor cells significantly decline with age (Figure 1A). To determine whether this decrease in proliferating neural stem and progenitor cells correlates with levels of TGF-β1, we isolated hippocampi from young (2–4 mo) and old (22–24 mo) male C57BL6/J mice. *Tgfb1* mRNA expression was examined by qRT-PCR, and TGF-β1 protein levels were analyzed both by ELISA in tissue lysates and via immunofluorescence in whole tissue sections. As shown in Figure 1B-1E, TGF-β1 became elevated with age in the murine hippocampus. Confirming a broad age-related increase of TGF-β1, this cytokine also became elevated in old blood serum, as assayed by both ELISA and Western blotting (Supplemental Data Figure 1A and [6]), and old skeletal muscle (Supplemental Data Figure 1B, 1C and [28].

TGF-β1 expression has been reported to rise with age in the subventricular zone of the forebrain [34, 40, 41], contributing to a decline in SVZ neurogenesis. In contrast, GDF11, which like TGF-β1 signals through ALK5/TGFBR2 receptor complex and pSmad2/3, has been suggested to enhance SVZ neurogenesis [9]. To investigate and reconcile the age-specific expression of multiple TGF-β family members in skeletal muscle and hippocampus, we performed mRNA and protein analysis. These results confirmed an age-specific increase in TGF- β1 in muscle and revealed an increase in the hippocampal stem cell niche (Supplemental Figure 1B–1C and Figure 1B–1E). Interestingly, however, *Tgfb2* mRNA did not change with age in myofibers, while *Tgfb3* and *Gdf11* – other TGF-β family ligands that signal through SMAD2/3 – were not expressed at detectable levels (Supplemental Figure 1D, 1H, 1I). In addition, qRT-PCR confirmed an increase in *Tgfb2* and *Tgfb3* mRNA levels in aged hippocampi, while *Gdf11* was expressed in hippocampi but did not change with age (Supplemental Figure 1E–1G).

**Figure 2:**
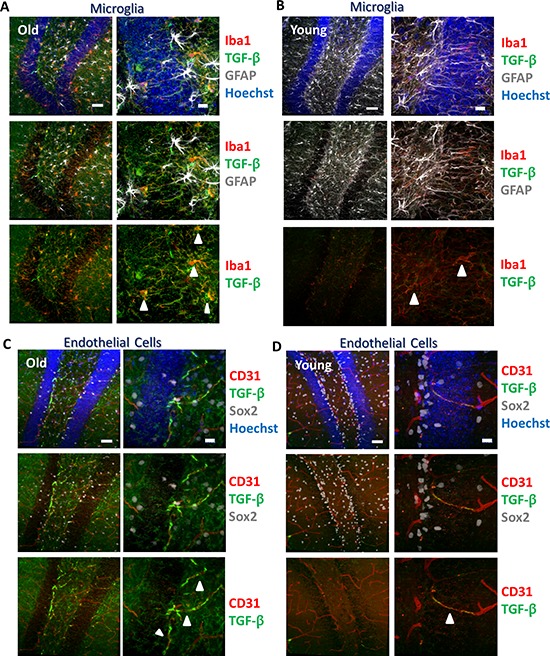
TGF-β is expressed by microglia and endothelial cells **A.** Immunofluorescence was performed on perfused old brain tissue sections for TGF-β1,2,3 (green), the microglial marker Iba1 (red), and astrocyte marker GFAP (gray), with Hoechst (blue) labeling all nuclei. Representative low and high magnification images are shown. Scale bars = 50 μM. Arrows indicate areas of TGF-β and Iba1 colocalization. **B.** Immunofluorescence was performed on perfused young brain tissue sections for TGF-β1,2,3 (green), Iba1 (red), and GFAP (gray), with Hoechst (blue) labeling all nuclei. Representative low and high magnification images are shown. Scale bars = 50 μM. Arrows indicate areas of TGF-β and Iba1 colocalization. **C.** Immunofluorescence was performed on perfused old brain tissue sections for TGF-β1,2,3 (green), endothelial marker CD31 (red), and Sox2 (gray), with Hoechst (blue) labeling all nuclei. Representative low and high magnification images are shown. Scale bars = 50 μM. Arrows indicate areas of TGF-β and CD31 colocalization. **D.** Immunofluorescence was performed on perfused young brain tissue sections for TGF-β1,2,3 (green), CD31 (red), and Sox2 (gray), with Hoechst (blue) labeling all nuclei. Representative low and high magnification images are shown. Scale bars = 50 μM. Arrow indicates area of TGF-β and CD31 colocalization.

To assess the cellular source of elevated TGF-β production, additional immunostaining of astrocytes, microglia, and endothelial cells was performed using an antibody that reacts with TGF-β1-3. We found that in the hippocampus, microglia and endothelial cells, but not astrocytes, expressed TGF-β in both old and young dentate gyri (Figure 2A-2D), suggesting they are sources of the age-associated increases in TGF-β.

**Figure 3:**
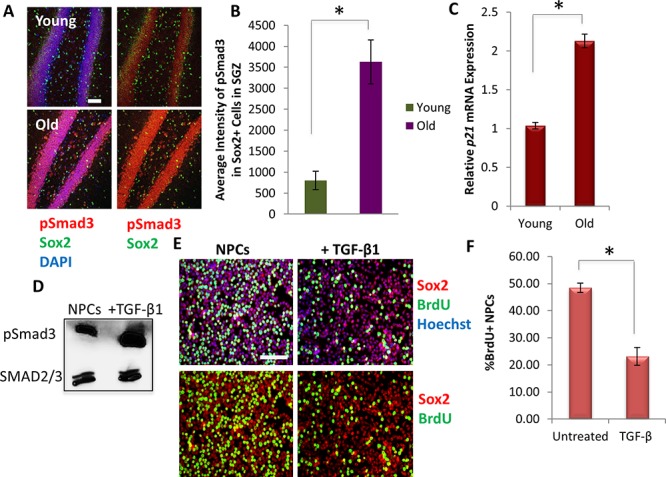
Downstream effectors of TGF-β signaling increase with age in mice hippocampi and inhibits neural progenitor cell proliferation **A.** Immunofluorescence staining for Sox2 (green) and pSmad3 (red) was performed on young and old brain tissue sections (*n* = 4 young, 6 old), with DAPI (blue) labeling all nuclei. Representative images are shown. Scale bar = 50 μM. **B.** MetaXpress image quantification was developed to calculate the average pixel intensity specifically in Sox2+ neural stem and progenitor cells in the SGZ of the dentate gyrus. Significant differences were identified by Student's *t*-tests (two-tailed) (*p < 0.001). Error bars indicate standard error of the mean (*n* = 4 young, 6 old). **C.** qRT-PCR quantification of *p21* mRNA expression from young and old hippocampi. The relative average expression level was normalized by *GAPDH* and presented relative to young hippocampi. Significant differences were identified by Student's *t*-tests (two-tailed) (*p < 0.003), and error bars indicate standard error of the mean (*n* = 3 young, 3 old biological replicates per group). **D.** Immunoblotting analysis of a downstream effector of TGF-β, pSmad3, from NPCs cultured in growth medium in the presence or absence of TGF-β1 (50 ng/mL) for 30 minutes. pSmad3 signaling is induced through TGF-β1, as compared with levels of total SMAD2/3. **E.** Functional validation of increasing TGF-β1. NPCs were cultured in growth medium (DMF12 + N2 + 10 ng/mL FGF-2) in the presence or absence of TGF-β1 (100 ng/mL) for 24 hrs. A 2 hour BrdU (10 μM) pulse was performed before cell fixation to label proliferating cells. Staining was conducted for BrdU (green) and Sox2 (red), with Hoechst (blue) labeling all nuclei. Representative images are shown. Scale bar = 100 μM. **F.** Proliferation of NPCs was quantified by cell scoring in 25 random fields of each condition using an automated imager and MetaXpress cell scoring software. Results are displayed as the mean percent of BrdU+ proliferating NPCs +/−SD, respectively. Significant differences were identified by Student's *t*-tests (two-tailed) (*p < 0.0003), and error bars indicate standard error of the mean (*n* = 4 biological replicates).

To confirm and build upon these results, we analyzed downstream pSmad signaling in young versus aged neural stem cells *in vivo*. As shown in Figure 3A (and quantified in 3B), the levels of pSmad3 were up-regulated in aged compared to young Sox2+ neural stem and progenitor cells in the SGZ. Furthermore, TGF-β1 is a known inhibitor of cell proliferation [26, 27, 35, 37], and accordingly qRT-PCR revealed that *in vivo* hippocampal expression of *p21*, a known down-stream target of TGF-β1/pSmad pathway that inhibits cell cycle progression [37, 42], was elevated with age (Figure 3C). To probe whether the age-associated increase in TGF-β expression may be functionally important for neurogenesis, TGF-β1 ligand was added to hippocampal-derived Sox2+ neural progenitor cell (NPCs) cultures and observed to both upregulate pSmad3 (Figure 3D) and decrease cell proliferation, as assayed by reduced BrdU uptake (Figure 3E-3F). These data are the first to profile the age-specific expression of TGF-β family ligands in the neurogenic and myogenic tissue niches. Furthermore, these data are the first to demonstrate that the levels of TGF-β1 transcript and protein and *p21* transcript increase with age in the hippocampus and particularly in microglia, and that SMAD3 phosphorylation increases in resident Sox2+ neural stem and progenitor cells of the old hippocampus.

### Simultaneous systemic enhancement of hippocampal neurogenesis and myogenesis in old mice

**Figure 4:**
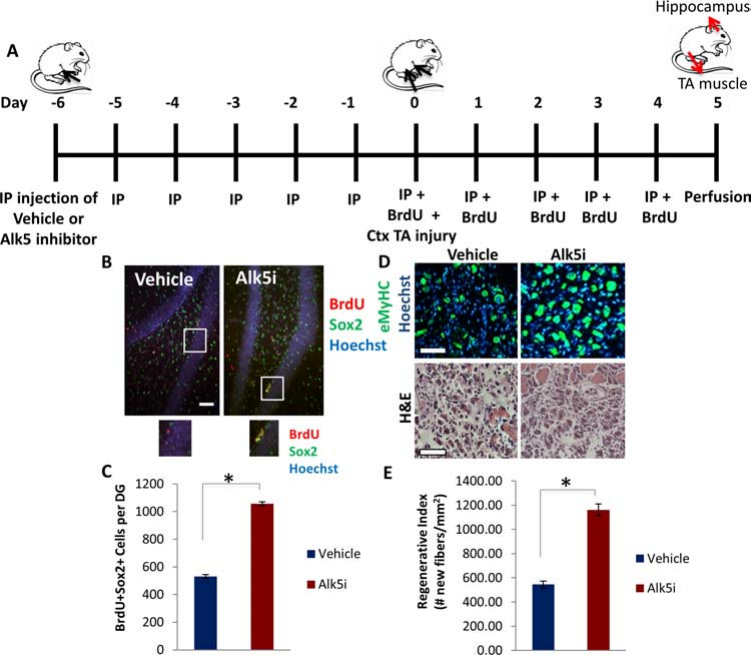
Simultaneous partial rescue of hippocampal neurogenesis and myogenesis in aged mice through systemic *in vivo* inhibition of TGF-β **A.** Schematic of Alk5 inhibitor administration. Aged (2 year old) mice received IP injections of TGF-β type I receptor kinase Alk5 inhibitor (Alk5i), or vehicle control, once daily for 11 days (Day −6 through Day 4) and were sacrificed on Day 5. Tibialis anteriors (TAs) were injured with cardiotoxin on Day 0, and mice began receiving daily IP injections of BrdU through Day 4. TAs were collected on Day 5, 5 days post injury (5 dpi). **B.** Following perfusion and PFA fixation, brain sections of Alk5 inhibitor or vehicle treated mice (*n* = 4 per group) spanning the entire hippocampus were immunostained for BrdU (red) and Sox2 (green), with Hoechst (blue) labeling cell nuclei. Representative images are shown. Scale bar = 50 μM. **C.** Quantification by stereology of BrdU+Sox2+ cells per dentate gyrus. As shown, Alk5 type I receptor kinase inhibitor increases the average number of BrdU+Sox2+ cells in the dentate gyrus. Significant differences were identified by Student's *t*-tests (two-tailed) (*p < 5 × 10^−7^). Error bars indicate standard error of the mean (*n* = 4 young, 4 old biological replicates) **D.** TA muscle sections collected 5 days post injury were immunostained for eMyHC (green), with Hoechst (blue) labeling cell nuclei. Representative images show newly regenerating myofibers in injured muscle sections. Hematoxylin and eosin staining of injury sites are also shown. Scale bars = 100 μM. **E.** Regenerative index quantifies the number of newly formed eMyHC+ myofibers per square millimeter in the injured area. Systemic administration of the Alk5 inhibitor enhances old muscle regeneration after injury, as displayed by the mean regenerative index per group. Significant differences were identified by Student's *t*-tests (two-tailed) (*p < 0.0002), and error bars indicate standard error of the mean (*n* = 4 biological replicates per group).

The conserved increase in TGF-β1/pSmad3 signaling within muscle and brain stem cell niches with age suggested that stem cell responses could be enhanced in both tissues by attenuating the intensity of this pathway, which would both validate our conclusions and offer translational potential for rejuvenating multiple tissues in the same organism with a single therapeutic intervention. Accordingly, a small molecule drug pharmacological inhibitor of the TGF-β receptor I kinase (Alk5), was added to cultured NPCs, where it was found to down-modulate pSmad2 and pSmad3 levels (Supplemental Figure 2A, quantified in B). *In vivo*, this drug was administered systemically via intraperitoneal (IP) injection into aged mice (24–26 months old) once daily for 11 days. In mice perfused on day 12, a robust decrease in pSmad2/3 levels was observed in the hippocampus (Supplemental Figure 2C-2F). To assess the ability of the Alk5 inhibitor to enhance regeneration of multiple tissues in the same animal, muscle was injured by cardiotoxin one week after the start of Alk5 inhibitor administration (day 0 of schematic Figure 4A), and tissue regeneration was analyzed at 5 days post-injury (Figure 4A). In parallel, we quantified hippocampal neurogenesis – i.e. the numbers of BrdU+Sox2+ cells in tissue sections across the hippocampus – in the same animal cohorts (Figure 4B-4C). Very interestingly, both neurogenesis and myogenesis were significantly enhanced in the aged mice treated with Alk5 inhibitor, compared to the animals receiving control buffer (Figure 4B-4E). In brain this was evident from a two-fold increase in the numbers of proliferating, Sox2+ neural stem and progenitor cells within the hippocampal dentate gyrus (Figure 4B-4C). In muscle we observed enhanced de novo myofiber formation 5 days post-injury as assayed by quantifying the numbers of eMyHC+ fibers with centrally located nuclei (Figure 4D, 4E). These results confirmed the pro-myogenic effects of Alk5 inhibitor [6, 43] and revealed that systemic administration of an attenuator of the TGF-β/pSmad3 pathway simultaneously rejuvenated skeletal myogenesis and hippocampal neurogenesis in the same old animal.

### Rejuvenation of myogenesis and neurogenesis by genetic attenuation of TGF-β signaling

**Figure 5:**
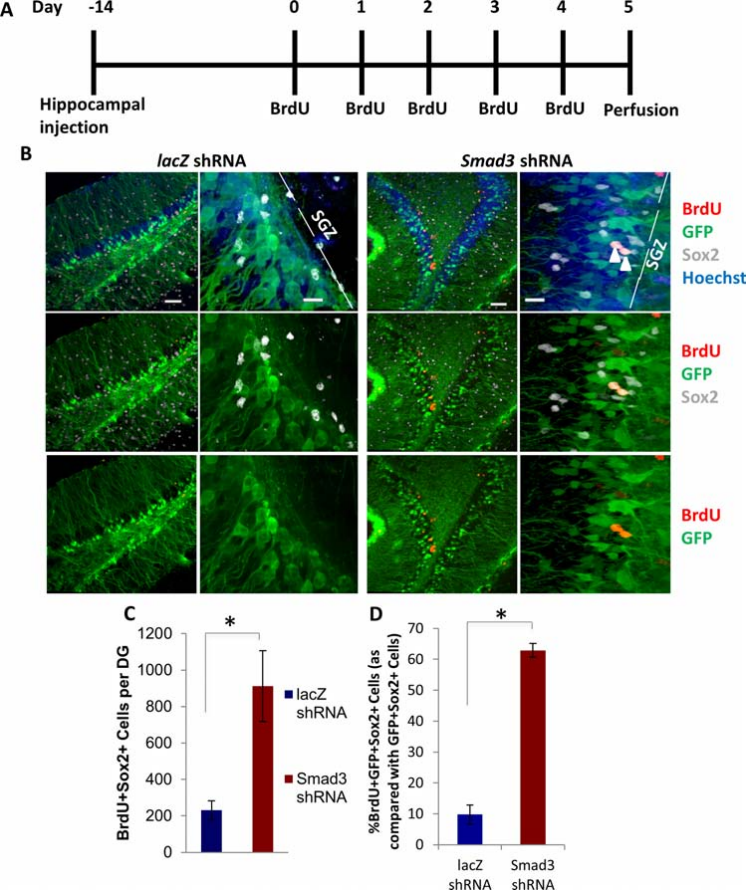
Rescue of neurogenesis in aged hippocampi by *in vivo* genetic inhibition of *smad3* **A.** Schematic of stereotaxic lentiviral injection experiment. Aged (18 month old) mice received stereotaxic injections into hippocampi (coordinates from bregma: AP: −2.12, ML: +/−1.5, VD: −1.55) of lentiviral vectors encoding shRNA against either *Smad3* or *lacZ*. Mice were allowed to recover for 14 days, followed by daily BrdU (50 mg/kg) intraperitonial injections for 5 days, then analysis. **B.** Brain sections of *lacZ* or *Smad3* shRNA injected mice (*n* = 5 *lacZ* shRNA lentivirus, 4 *Smad3* shRNA) spanning the entire hippocampus were immunostained with GFP (green), BrdU (red), and Sox2 (gray), with Hoechst (blue) labeling cell nuclei. Representative low and high magnification images are shown. Scale bars = 50 μM. Arrows in high magnification image indicate transduced, proliferating NPCs. **C.** Quantification of the mean number of BrdU+Sox2+ cells per dentate gyrus shows an increase in proliferating NPCs in aged animals expressing anti-*Smad3* shRNA. Significant differences were identified by Student's *t*-tests (two-tailed) (*p < 0.05). Error bars indicate standard error of the mean (*n* = 5 *lacZ* shRNA, 4 *Smad3* shRNA brains). **D.** Quantification of the mean percentage of BrdU+GFP+Sox2+ proliferating NPCs as compared with BrdU-GFP+Sox2+ NPCs in shRNA injected brains demonstrates an increase in proliferating neural progenitor cells due to shRNA-*Smad3* transduction as compared with shRNA-*lacZ* control mice. Significant differences were identified by Student's *t*-tests (two-tailed) (*p < 4 × 10^−7^), and error bars indicate standard deviation (*n* = 3).

To confirm these findings using independent experimental approaches, we inhibited TGF-β signaling using a lentivirally-encoded shRNA we developed against *Smad3*. The efficacy of this shRNA was confirmed in transduced mouse muscle progenitor cells *in vitro* via western blotting and in mouse neural progenitor cells *in vitro* via qRT-PCR (Supplemental Figure 3A-3B). After a single stereotaxic hippocampal injection of a lentiviral vector encoding GFP plus the shRNA against *Smad3* – or a control shRNA against *LacZ* – into 24 month old mice, animals were allowed to recover for 2 weeks, followed by five consecutive days of BrdU administration (Figure 5A). As shown in Figure 5B–5C, compared to control shRNA lentiviral transduction, the numbers of Sox2+ proliferating cells (quantified in the GFP+ region of tissue sections throughout the entire hippocampus) were significantly increased after a single injection of shRNA to *Smad3*. There was also a significantly higher proportion of GFP+Sox2+ cells that were also BrdU+ (Figure 5D), confirming lentiviral delivery of shRNA against *Smad3*, but not control shRNA, enhanced neural progenitor cell proliferation. Neurogenesis was thus significantly enhanced by the inhibition of *Smad3* signaling in the local niche of neural stem cells in 2 year old mice (analogous to 80 year old humans), demonstrating progress in rejuvenating neurogenesis in very old mice, and complementing reports on the enhancement of neurogenesis through increase of Wnt-mediated signaling in 13 month old mice [44].

To analyze analogous effects on downstream type 2b Doublecortin expressing (DCX+) transit amplifying cells, another cohort of animals was mitotically labeled (with EdU) for 5 consecutive days and studied 5 days after the final injection to detect the cells that had progressed towards neuronal commitment [45]. There was a substantial increase in the total number of EdU+DCX+GFP+ type 2b cells (Supplemental Figure 4A-4C), demonstrating that not only the proliferation of neural stem and progenitor cells but the generation of neuronally committed cells is enhanced in old mice administered with *Smad3* shRNA.

To further compare molecular conservation of tissue aging between brain and muscle, we also performed a single administration of the lentiviral vector encoding shRNA against *Smad3* into injured hind leg muscle of 24 month old mice, simultaneously with cardiotoxin to induce muscle injury [19, 46]. Significant enhancement of old muscle repair, based on the numbers of de-novo formed muscle fibers that were quantified throughout the entire injury site [19, 47], was observed in the 24 month old mice injected with the shRNA to *Smad3*, compared to the control shRNA (Supplemental Figure 4D, 4E), and consistent with our prior report [35].

**Figure 6:**
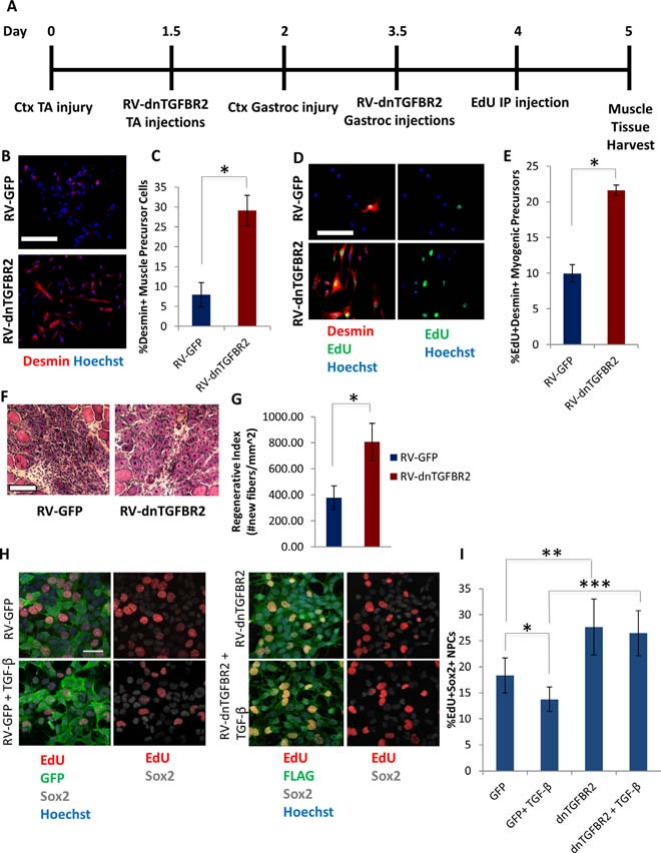
Rescue of myogenesis in aged muscle and enhanced NPC proliferation by *in vivo* genetic inhibition of TGF-β receptor II **A.** Schematic. Aged (18 month old) mice TA muscle was injured with cardiotoxin on Day 0, followed by injection of dnTGFBR2 retrovirus into injury sites 36 hours later, on Day 1.5. On Day 2 gastrocnemius muscle were injured, followed by injection of dnTGFBR2 retrovirus at sites of injury 36 hours later, on Day 3.5. Mice received IP injection of EdU 12 hours before muscle tissue was harvested. **B.** Myogenic precursor cells isolated 3 days post-injury from gastrocnemius muscle were stained for Desmin (red), with Hoechst (blue) labeling all cell nuclei. Representative images are shown. Scale bar = 50 μM. **C.** Quantification of the mean percentage of Desmin+ precursor cells. Significant differences were identified by Student's *t*-tests (two-tailed) (*p < 0.03). Error bars indicate standard error of the mean (*n* = 3 biological replicates). **D.** Myogenic precursor cells isolated 3 days post-injury from gastrocnemius muscle were stained for Desmin (red) and EdU (green), with Hoechst (blue) labeling all cell nuclei. Representative images are shown. Scale bar = 50 μM. **E.** Quantification of the mean percentage of proliferating myogenic precursor cells isolated from muscle that received dnTGBR2 or GFP. Significant differences were identified by Student's *t*-tests (two-tailed) (*p < 0.02), and error bars indicate standard error of the mean (*n* = 3 biological replicates per group) **F.** Hematoxylin and eosin of muscle TAs isolated 5 days post-injury and sectioned in a cryostat at 10 μM. Representative images shown. Scale bar = 100 μM. **G.** Five days post-injury regeneration of old mice tibialis anterior muscle, which received dnTGFBR2 or GFP retrovirus, was quantified from muscle sections (10 μM sections throughout sites of injury) and is presented as the mean number of newly regenerated myofibers per square millimeter of injury site. Error bars indicate standard error of the mean, (*n* = 3 mice per group). Significant differences were identified by Student's *t*-tests (two-tailed) (*p < 0.01). **H.** NPCs were transduced with dnTGFBR2 or GFP and cultured for one week, followed by 16 hour incubation in growth medium in the presence/absence of 10 ng/mL TGF-β1. Cells were pulsed with EdU (30 μM) for 4 hours before fixation to label proliferating cells. Cells were stained for GFP or FLAG (green), EdU (red), and Sox2 (gray), with Hoechst (blue) labeling cell nuclei. Representative images are shown. Scale bar = 100 μM. **I.** Proliferation of NPCs was quantified by cell scoring in 36 random fields of each condition using an automated imager and MetaXpress cell scoring software. Results are displayed as the mean percent of EdU+Sox2+ proliferating NPCs +/−SD, respectively. Significant differences were identified by Student's *t*-tests (two-tailed) (*p < 9 × 10^−10^, **p < 3 × 10^−24^, ***p < 4 × 10^−16^). Error bars indicate standard deviation (*n* = 36 technical replicates). The experiment was replicated 4 times.

We further corroborated our findings by introducing a dominant negative form of the TGF-β1 receptor (dnTGFBR2, known to inhibit pSmad2/3 levels [48]). Expression of the dnTGFBR2 in neural progenitor cells and downstream inhibition of pSmad3 were confirmed by western blotting (Supplemental Figure 5A, 5B). Retroviral vectors encoding dnTGFBR2 or GFP as a control were injected into CTX injured muscle one day after injury, when resident muscle stem cells are breaking quiescence and entering the cell cycle [1, 19] (Figure 6A). Three days after injury, muscle was harvested and assayed for proliferating (EdU+) myogenic cells. Compared to the aged tissue injected with control vector, in old muscle administered with dnTGFBR2 retroviral vector there was a marked increase in the percentage of desmin+ muscle precursor cells (Figure 6B, 6C) and a significant increase in proliferating desmin+EdU+ myogenic cells (Figure 6D, 6E). We also examined muscle tissue 5 days after injury and observed that repair of old damaged muscle *in vivo* was robustly enhanced by dnTGFBR2, as assayed by the replacement of the injury with the newly formed myofibers and reduction in scarring (Figure 6F, 6G). These results extend our previous work [6] to *in vivo* studies and interestingly suggest that retroviral delivery of dnTGFBR2 rejuvenated the myogenic capacity of old muscle stem cells already responding to tissue injury, suggesting that old muscle repair can be enhanced after the time of breakage of quiescence (e.g. by enhancing the proliferation of the few old muscle stem cells that have entered cell cycle). Importantly, these data confirm that canonical TGF-β1/pSmad signaling is involved in the age-imposed inhibition of myogenesis.

To further validate the cross-tissue conservation of TGF-β1 signaling with aging, hippocampal neural progenitor cells were transduced with dnTGFBR2-encoding retrovirus and assessed for proliferation. As shown in Figure 6H-6I, dnTGFBR2 increased neural progenitor proliferation in their (FGF-2-containing) growth medium as compared to GFP control, based on EdU incorporation. Western blotting analysis showed that there was relatively high pSmad3 signaling in NPCs in growth medium even in the absence of exogenous TGF-β1, which was diminished upon dnTGFBR2 retroviral transduction (Supplemental Figure 5A). Furthermore, TGF-β1 (10 ng/mL) addition to growth medium overnight inhibited proliferation of GFP+ NPCs, but did not affect dnTGBR2+ NPCs (Figure 6H, 6I). Collectively, these results including both pharmacological and genetic intervention suggest that TGF-β1/pSmad2/3 signaling increases with age in both myogenic and neurogenic niches of tissue stem cells and that attenuation of this pathway partially rescues myogenesis and neurogenesis.

### High TGF-β levels increase B2M expression in aged neural and muscle stem cell niches

The developmental origins and properties of muscle and brain (and their respective stem cells) are quite different, and we therefore next focused on potential underlying molecular mechanisms that may be regulated by the pleiotropic TGF-β/pSmad3 pathway and thus shared between such adult organ systems. The TGF-β/pSmad3 pathway exerts complex and sometimes reciprocal effects on cellular and tissue responses, including the rate of cell cycle progression and the anti- versus pro-inflammatory role in regulating immune responses [29]. Thus, we next examined the effects of the age-specific elevated TGF-β1 on tissue inflammation by assaying the levels of MHC class I gene expression, which is known to directly associate with and to promote inflammation in multiple tissues including in brain and muscle pathologies [40, 49, 50]. B2M is the invariant chain of the MHC class I protein complex, and its expression is regulated similarly to the variable/polymorphic MHC class I genes [51–53].

**Figure 7:**
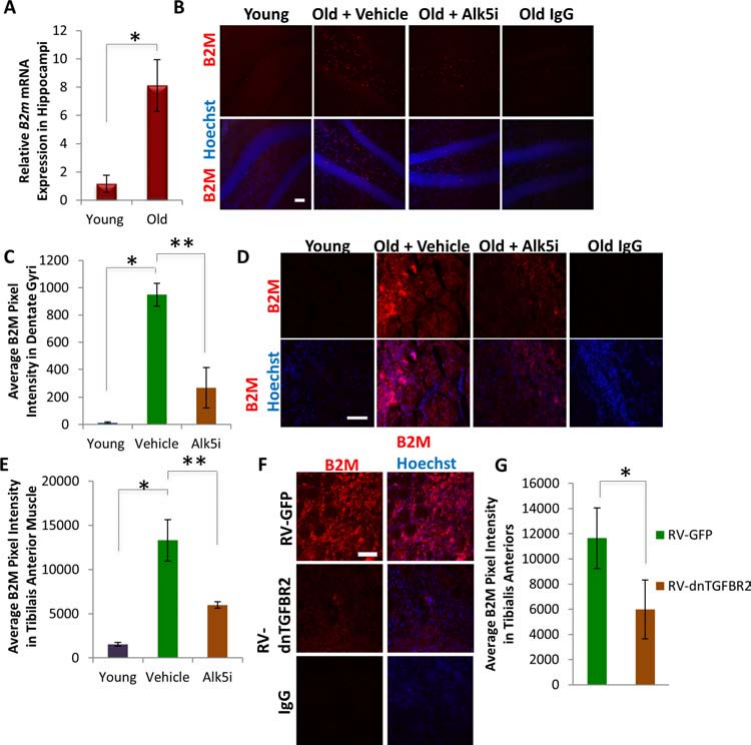
B2M levels decrease in neurogenic and myogenic niches following systemic or local attenuation of TGF-β signaling **A.** qRT-PCR quantification of *B2m* mRNA expression from young and old hippocampi. The relative average expression level was normalized by *GAPDH* and presented relative to young hippocampi. Significant differences were identified by Student's *t*-tests (two-tailed) (*p < 0.04), and error bars indicate standard error of the mean (*n* = 4 old, 4 young). **B.** Brain sections spanning the hippocampus from young mice (2 month), as well as Alk5 inhibitor or vehicle treated old (24 month) mice, were immunostained for B2M (red), with Hoechst (blue) labeling all cell nuclei. Representative images are shown. Scale bar = 50 μM. **C.** Average B2M pixel intensity was quantified using MetaXpress software, and significant differences were identified by Student's *t*-tests (two-tailed) (*p < 3 × 10^−6^, **p < 0.0001). Error bars indicate standard error of the mean (*n* = 5 young, 3 old + Alk5i, 3 old + vehicle biological replicates (mice), with *n* = 12 technical replicates per mouse) **D.** Tibialis anterior muscle collected 5 days post-injury were immunostained for B2M (red), with Hoechst (blue) labeling all cell nuclei. Representative images are shown. Scale bar = 100 μM. **E.** Average B2M pixel intensity of 10 μM tibialis anterior muscle sections throughout sites of injury were quantified using MetaXpress software. Significant differences were identified by Student's *t*-tests (two-tailed) (*p < 7 × 10^−7^, **p < 5 × 10^−5^), and error bars indicate standard error of the mean (*n* = 3 biological replicates per group (mice), with *n* = 12 technical replicates per mouse) **F.** TA muscles collected 5 days post injury were immunostained for B2M (red), with Hoechst (blue) labeling all cell nuclei. Representative images are shown. Scale bar = 100 μM. **G.** Average B2M pixel intensity of TA muscle sections throughout sites of injury were quantified using MetaXpress software. Significant differences were identified by Student's *t*-tests (two-tailed) (*p < 0.002). Error bars indicate standard error of the mean (*n* = 3 biological replicates per group (mice), with *n* = 12 technical replicates per mouse).

We compared the B2M expression levels as an indicator of inflammatory response in young and old mice treated with vehicle control, and old mice treated with the Alk5 inhibitor (Figure 4). B2M levels were extremely low in young brain and muscle, consistent with prior work [30], but increased significantly with aging (Figure 7A-7E). Importantly, when TGF-β signaling was attenuated *in vivo* by the Alk5 inhibitor, B2M levels were significantly diminished both in old brain (Figure 7B, 7C) and old muscle (Figure 7D, 7E). These results demonstrate that B2M becomes upregulated with aging in multiple tissues (suggesting an increase in inflammation) and that down-modulation of TGF-β signaling, which rejuvenates myogenesis and neurogenesis, normalizes B2M in myogenic and neurogenic regions to their young levels, suggesting attenuation of inflammation. As further support of this conclusion, the levels of B2M were also significantly reduced in regenerating regions of the old muscle administered with the dnTGFBR2, as compared to tissue administered with control GFP virus (Figure 7F, 7G). Furthermore, addition of low concentrations of TGF-β1 (0–5 ng/mL) to immune cells, specifically BV2 cells – a microglia cell line – did not affect B2M levels, as assayed by pixel intensity and percent area staining of B2M (Supplemental Figure 6A-6C). In contrast, high TGF-β1 (50 ng/mL) levels induced a significant increase in B2M expression. Thus, physiologically young levels TGF-β1 does not induce B2M, but increased TGF-β1 does.

## DISCUSSION

Collectively, these data are the first to suggest that similar molecular mechanisms acutely yet reversibly inhibit the capacity of stem cells in old muscle and in the hippocampus of the aged brain to contribute to differentiated tissue. This study specifically focused on age-imposed misregulation of the TGF-β/pSmad3 signaling pathway, and cross-tissue conservation was clearly identified. Confirming these conclusions and demonstrating clear therapeutic significance, systemic administration of an Alk5 Type I receptor kinase inhibitor simultaneously improved repair of skeletal muscle and enhanced hippocampal neurogenesis in 2-year old mice, suggesting promising strategies for combating multiple age-related degenerative disorders, which are known to contribute to the loss of a person's agility, mobility, memory, learning, and independence. These results show that excessive TGF-β/pSmad3 signaling induces inflammation in muscle and brain, as indicated by increased B2M expression. Systemic inhibition of the TGF-β type 1 receptor enhanced both neurogenesis and myogenesis and normalized B2M levels. An increase in inflammation resulting in part from pathologically high TGF-β1 levels may contribute to the broad decline in maintenance and repair of old tissues by their dedicated stem cells, in addition to up-regulation of p21 and other cell cycle inhibitors known to occur in skeletal muscle [1, 19] described here for the hippocampus.

Independent genetic and pharmacological approaches to attenuate TGF-β/pSmad3 signaling drove parallel enhancements of neurogenesis and myogenesis, thus providing important cross- validation of these results and conclusions. While local tissue delivery of shRNA against *Smad3* most likely inhibited TGF-β/pSmad3 signaling directly in muscle or brain, the systemic administration of Alk5 inhibitor could function both directly and potentially indirectly through attenuation of TGF-β pathway signaling in multiple tissues. Of note, both endothelial cells and microglia secrete TGF-β (Figure 2). TGF-β signaling is critical for the development of microglia and for the maintenance of their unique molecular signature in the adult CNS [54], thus suggesting that microglia may produce TGF-β for their own immunological functions in the brain in addition to signaling to other cells. In addition, microglia are known both to increase in number in the aged brain and also to develop a more inflammatory phenotype with higher expression of several cytokines, including TGF-β1 [55]. Finally, endothelium may also be a source of elevated TGF-β secretion in the hippocampus. Neural stem cells are known to be closely associated with blood vessels in a vascular niche, where endothelial cells are important regulators of neurogenesis that have been reported to promote self-renewal and prevent differentiation of NSCs through secretion of soluble factors [56]. Furthermore, the permissiveness of blood brain barrier increases in the aged and pathological brain [57], and the levels of TGF-β1 rise with age in circulation ([6], and Supplemental Figure 1), suggesting another means by which inhibitory amounts of this cytokine could reach the neurogenic SGZ. Finally, with potential significance to aging, TGF-β is known to be up regulated upon central nervous system damage, and such elevation is concurrent with the dramatic induction of MHC class I gene expression, as well as with such known phenotypes of the aged brain as activation of microglia and neuronal apoptosis [30].

Various studies have demonstrated that not just one signaling pathway, but a network of highly interactive pathways including Notch, Wnt, BMP, Shh, TNF-α, IGF, and IL-6 are affected by the aging process [1, 2, 46]. For example, Wnt signaling has been demonstrated to decrease in aged hippocampi and contribute to the decline of adult hippocampal neurogenesis [1, 44, 58, 59]. Furthermore, IL-6 becomes elevated in the old and especially at the sites of tissue damage [60], is secreted by activated microglia, and is known to promote inflammation in part via support of Th17 cells and up-regulation of MHC class I and class II gene expression [32, 61, 62]. Therefore, it is possible that the age-specific increase in TGF-β1 signaling may contribute to the excess of IL-6 or other inflammatory cytokines such as IL-17, and consequentially experimental attenuation of TGF-β1 may result in lower levels of inflammatory cytokines and normalized levels of MHC I proteins. The detailed understanding of the age-specific cytokine interplay in the neurogenic and myogenic niches would be interesting to uncover in future comprehensive studies.

In summary, this work improves our understanding of the aging of tissue stem cells, reveals a molecular conservation of the aging process between muscle and hippocampal region of brain, and suggests novel clinical strategies for the simultaneous rejuvenation of myogenesis and neurogenesis in old mammals.

## EXPERIMENTAL PROCEDURES

### Animals

Young (2–3 month old) and old (18–24 month old) C57BL6/J male mice were purchased from the Jackson Laboratory and the NIH. The animal experimental procedures were performed in accordance with the Guide for Care and Use of Laboratory Animals of the National Institutes of Health, and approved by the Office of Laboratory Animal Care, UC Berkeley.

For each experiment on aged (18–24 month old) mice, an n of at least 7 per group would be used initially. If aged mice died due to surgery or age-related health issues, a minimum of *n* = 3 remaining mice were analyzed and assessed for statistical significance (described in statistical analysis below). Mice of the same genetic strain and age were assigned to groups at random.

### Cell culture

Primary rat neural progenitor cells isolated from the hippocampi of female Fisher 344 rats (Charles River) were cultured in growth medium (DMEM/F12 (Life Technologies) containing N2 supplement (Life Technologies) and 10 ng/mL FGF-2 (PeproTech)) on laminin (Roche) and polyornithine (Sigma) coated tissue culture plates, with subculturing on reaching 80% confluency using Accutase (Phoenix Flow Systems), as previously described [63].

Primary mouse neural progenitor cells were isolated froom C57BL6/J male mice (Charles River) as previously described [64]. Cells were cultured in growth medium (Neurobasal A (Gibco) containing B27 supplement (Gibco), Glutamax-1 supplement (Gibco), 20 ng/mL FGF-2 (Peprotech), and 20 ng/mL EGF (PeproTech)) on Poly-d-Lysine (Sigma) and Laminin (Roche) coated tissue culture plates, with subculturing on reaching 80% confluency using Accutase (Phoenix Flow Systems).

Primary mouse muscle progenitor cells (myoblasts) were isolated as previously described [65] and cultured and expanded in growth medium containing: Ham's F-10 (Gibco), 20% Bovine Growth Serum (Hyclone), 5 ng/mL FGF-2 (PeproTech) and 1% penicillin-streptomycin on Matrigel (BD Biosciences) coated plates (1:300 Matrigel: PBS), with subculturing on reaching 80% confluency using 0.5% Trypsin (Sigma Aldrich) diluted in PBS.

Progenitor cells were tested for miscroplasma contamination at the UC Berkeley Stem Cell Core Facility and using Hoechst DNA stain.

Primary Muscle stem cells were isolated from male C57BL6/J mice as described below and cultured in DMEM (Life Technologies) with 5% serum from the same age mouse on Matrigel (BD Biosciences) coated plates (1:100 Matrigel:PBS) overnight before cell fixation with 70% cold ethanol.

BV2 microglia (gift of Wyss-Coray lab, Stanford), were cultured in DMEM (Gibco) + 10% Fetal Bovine Serum (Hyclone), with subculturing on reaching 80% confluency using 0.5% Trypsin (Sigma Aldrich) diluted in PBS.

### *In vitro* validation of TGF-β1 signaling and proliferation assay

Primary rat NPCs were cultured in growth medium (DMF12 + N2 + 10 ng/mL FGF-2) as described above. Cells were cultured at a density of 200,000 cells per well of a 6-well culture slide in the presence/absence of TGF-β1 (50 ng/mL) (HumanZyme, Inc.) for 30 minutes, followed by a PBS wash and cell scraping into RIPA buffer for western blot analysis as described below. rNPCs were also cultured at a density of 80,000 cells per well of an 8-well chamber slide in growth medium plus the presence/absence of TGF-β1 (100 ng/mL) for 24 hours. NPCs were pulsed for 2 hours with 10 μM BrdU (Sigma Aldrich) before cell fixation with 70% cold ethanol for immunocytochemistry analysis as described below.

BV2 microglia were cultured in growth medium as described above. For experimental treatment, cells were then cultured at a density of 80,000 cells per well of an 8-well chamber slide in DMEM plus the presence of lipopolysaccharide (LPS) (100 ng/mL) (Invivogen) and IFN-γ (20 ng/mL) (Peprotech, Inc.) for 24 hours in order to stimulate BV2s to an inflammatory activated phenotype. TGF-β1 (PeproTech, Inc.) was concurrently added to cells for 24 hours at 0–50 ng/mL to assess the inflammatory response. After 24 hours, cells were fixed with 4% paraformaldehyde for immunocytochemistry analysis as described below.

### Dissection and preparation of murine hippocampi for RNA or protein analysis

Young or old mice were anesthetized and perfused with 20 mL saline, followed by dissection of hippocampi. For RNA extraction, tissue was placed in 1 mL Trizol (Invitrogen) and homogenized, followed by chloroform extraction as previously described [63]. For protein extraction, hippocampi tissue was homogenized in RIPA buffer (50 mM Tris, 150 mM NaCl, 1% NP40, 0.25% sodium deoxycholate and 1 mM EDTA, pH 7.4) containing 1X protease inhibitor (Roche), 1 mM Phenylmethylsulfonyl fluoride (PMSF), 1 mM sodium fluoride and 1 mM sodium orthovanadate. The tissue debris was spun at 10K rpm for 5 min at 4C, and supernatant containing protein extract snap frozen with dry ice.

### RNA extraction, RT-PCR and real-time PCR

Total RNA was extracted from primary neural progenitor cells or young and old murine hippocampi using Trizol reagent (Invitrogen) according to manufacturer's instructions. 1 ug of total RNA was used for cDNA synthesis with Olig D_t_ primers (Invitrogen). For real-time PCR amplification and quantification of genes of interest, an initial amplification using specific primers to each gene of interest (realtimeprimers.com) was done with a denaturation step at 95°C for 5 min, followed by 45 cycles of denaturation at 95°C for 1 min, primer annealing at 58°C for 30 s, and primer extension at 72°C for 15 s. Real-time PCR was performed using SYBR and an ABI PRISM 7500 Sequence Detection System (Applied Biosystems). Reactions were run in triplicate in three independent experiments. The geometric mean of housekeeping gene *GAPDH* was used as an internal control to normalize the variability in expression levels and were analyzed using the 2^−ΔΔCT^ method described [66].

### ELISA

The concentration of active TGF-β1 in blood serum or tissue protein lysate was determined using enzyme-linked immunoabsorbent assay (ELISA)-based cytokine antibody array (R&D), according to manual instructions.

### Immunocytochemistry

Mice were anesthetized and perfused with saline and 4% PFA. Brains were collected and placed in 4% PFA overnight for a post fixation, followed by dehydration in 30% sucrose/PBS at 4°C for 2 days. Brains were then sectioned at 40 μM using a vibratome and stored in a glycerol-based cryoprotectant at −20°C until further analysis by immunostaining. For non-BrdU/EdU staining, sections were washed 3 times for 15 minutes in TBS, followed by one hour blocking in a permeabilization/staining buffer, TBS++ (3% Donkey Serum and 0.25% Tween X-100 in TBS), then incubated with primary antibodies of interest (see Antibodies) for 72 hours. For secondary staining, sections were washed 3 times, 15 minutes each in TBS, followed by 2 hour incubation in donkey raised, fluorophore-conjugated, species-specific secondary antibodies (Jackson Immunoresearch) at 1:250 dilution in TBS++. Following secondary staining, sections underwent 3, 15 minute washes in TBS, with 4 μM Hoechst in the second wash. Finally, the sections were mounted on positively charged frosted slides, dried overnight and imaged with a prairie confocal microscope.

### BrdU *in vivo* labeling and immunostaining

Mice were intraperitoneally injected with BrdU (50 mg/kg of body weight, Sigma Aldrich) dissolved in saline to label mitotic cells. Sections were incubated in SSC/formamide at 65°C water for 2 hours, washed in TBS, followed by 2N HCl for 15 minutes. They were then placed in 2X Saline-Sodium Citrate (SSC) for 30 minutes, .1 mM Borate Buffer for 15 minutes, followed by 6, 15 minute washes in TBS, then blocked in a permeabilization/staining buffer, TBS++ for 2 hours. Sections were then incubated with αRat-BrdU (Abcam Inc. ab6326) and other antibodies (see Antibodies section) in TBS++ at 4°C for 72 hours. Secondary staining was done as described above.

### EdU *in vivo* labeling and immunostaining

Mice were intraperitoneally injected with EdU (50 mg/kg of body weight, Invitrogen) dissolved in phosphate-buffered saline to label mitotic cells. Brain sections were post-fixed with 4% PFA for 30 minutes after primary and secondary staining, and treated for EdU visualization using the Click-iT EdU kit (Invitrogen), as per the manual's instructions.

Muscle progenitor cells were fixed with cold, 70% ethanol and stained for αRabbit-Desmin and secondary staining as described below under muscle methods. Following secondary staining, cells were treated for EdU visualization using the Click-iT EdU kit (Invitrogen), as per the manual's instructions.

### Western blot analysis

Primary neural or muscle progenitor cells, or whole muscle tissue were lysed in RIPA buffer (50 mM Tris, 150 mM NaCl, 1% NP40, 0.25% sodium deoxycholate and 1 mM EDTA, pH 7.4) containing 1X protease inhibitor (Roche), 1 mM Phenylmethylsulfonyl fluoride (PMSF), 1 mM sodium fluoride and 1 mM sodium orthovanadate. The protein concentration was determined by Bradford assay (Bio-Rad). Lysates were resuspended in 1X Laemmli buffer (Bio-Rad), boiled for 5 minutes and separated on precast 7.5% or 4–15% TGX gels (Biorad). Primary antibodies were diluted in 5% non-fat milk in TBS + 0.1% Tween-20, and nitrocellulose membranes were incubated with antibody mixtures overnight at 4°C. HRP-conjugated secondary antibodies (Santa Cruz Biotech) were diluted 1:500 in 5% non-fat milk in TBS + 0.1% Tween-20 and incubated for 1 hour at room temperature. Blots were developed using Western Lightning ECL reagent (Perkin Elmer), and analyzed with Bio-Rad Gel Doc/Chemi Doc Imaging System and Quantity One software. Results of multiple assays were quantified using Applied Biosystems or Image J software. Pixel Intensity of bands of interest were normalized with pixel intensity of glyceraldehydes-3-phosphate dehydrogenase or β-actin.

### Antibodies

αRabbit-pSmad2 (Millipore AB3849), αRabbit-βactin (Cell Signaling #4967), αMouse-TGFβ1 (R&D MAB240), αMouse-TGFβ1,2,3 (R&D MAB1835), EdU Click-it kit (Invitrogen C10337 and C10338), αGuineyPig-DCX (Millipore AB2253), αGoat-Sox2 (Santa Cruz SC-17320), αRabbit-Desmin (Abcam ab32362), αMouse-embryonic myosin heavey chain (eMyHC) (Hybridoma Bank, clone F1.652), αChicken-GFP (Abcam ab13970), αRabbit-FLAG (Santa Cruz sc-807), αMouse-FLAG (Abcam ab18230), αRabbit-pSmad3 (Epitomics #1880-1), αRat-BrdU (Abcam ab6326), TGF-β1 ELISA kit (R&D DY1679), αMouse-Smad2/3 (Santa Cruz sc-133098), αRabbit-B2M (Abcam ab75853), αGoat-GapDH (Abcam ab9483), αRat-CD31 (BD Biosciences 550274), αGoat-Iba1 (Abcam ab5076), αRabbit-GFAP (Abcam ab7260).

### *In vitro* validation of Alk5 inhibitor in rNPCs

Following overnight rNPC culturing in growth medium at a density of 300,000 cells per well of a 6 well tissue culture plate, rNPCs were starved in a basal medium (lacking FGF-2) for 1 hour, then untreated, or treated with 1 ng/mL TGF-β1 for 1 hour in the presence/absence of 5 uM of Alk5 inhibitor, followed by a PBS wash and cell scraping to collect cells into RIPA buffer for western blot analysis.

### *In vivo* validation of Alk5 inhibitor

Aged (24 month old) C57BL6/J male mice were injected intraperitonially (IP) with TGF-β1 Type I Receptor Kinase Alk5 inhibitor 2-(3-(6-Methylpyridin-2-yl)-1*H*-pyrazol-4-yl)-1,5-naphthyridine (Enzo Life Sciences) diluted in sunflower seed oil to a concentration of 57.4 μM. Mice (*n* = 4) received 100 uL IP injections of the Alk5 inhibitor or vehicle control once daily for 11 days, and were perfused on the 12^th^ day. Tibialis Anterior muscle was injured with cardiotoxin after one week of IP injections, on day 7 (as described below in muscle methods). Additionally, on day 7 we began daily IP BrdU injections (50 mg/kg body weight). Four hours after receiving the fifth BrdU IP injection, the mice were perfused (on Day 12), and brains and TAs were collected for analysis.

### Lentiviral and retroviral vector construction, packaging, and purification

A DNA cassette encoding human U6 promoter-driven expression of shRNA against mouse *Smad3* (Gene ID: 17127) was constructed by PCR with flanking *Pac I* sites and, following restriction digestion and phenol/chloroform purification, ligated into the *Pac I* site of the pFUGW lentiviral vector. Five candidate sequences were tested for knockdown efficiency, and the most effective sequence (shSMAD 3.4 in Supplemental Table 1) was selected for experimental studies. Sequences for all shRNAs tested are provided in Supplemental Table 1. The control shRNA vector against *LacZ* was constructed previously [63]. PCR was performed with Phusion DNA Polymerase (New England Biolabs) under the following conditions: 98°C for 2 min, 30 cycles of 12 s at 95°C, 30 s at 65°C, and 25 s at 72°C, with a final extension step of 2 min at 72°C. Lentiviral and retroviral vectors were packaged and purified using standard methods as described [67].

A dominant negative TGFBR2 retroviral plasmid was obtained (Addgene, Cambridge, MA, http://www.addgene.org/12640/) and packaged and purified as previously described [68].

### *In vitro* validation of smad3 shRNA vector

Mouse myoblasts were cultured for 24 hours at a density of 100,000 cells per well of a 6 well tissue culture plate in 50% growth medium and 50% lentiviral supernatant (in DMEM + 10% FBS), packaged as previously described [68], followed by culturing in growth medium for a total of 72 hours. Cells were then washed once with PBS, scraped and collected into RIPA for western blot analysis.

mNPCs were plated at 200,000 cells per well of a 6 well tissue culture plate in growth medium, and transduced with lentivirus encoding shRNA to *Smad3* or *lacZ* at a multiplicity of infection (MOI) of 5 and cultured for two weeks. RNA was extracted with Trizol (Invitrogen) followed by qPCR to assess levels of *Smad3* (see Supplemental Table 1 for sequences).

### *In vivo* loss of function with shRNA

Aged (18 month) C57BL6/J male mice received lateral intrahippocampal injections of 1 μl of lentiviral solutions (LV-shRNA-Smad3-GFP or LV-shRNA-lacZ-GFP, 1–3 × 10^8 IU/mL) in PBS on day −14, on the right hemisphere hippocampus, at 0.25 uL per minute. The injection coordinates with respect to bregma were −2.12 anteriorposterior, −1.55 dorsoventral (from the dura), and 1.5 mediolateral (refer to Figure 5A). Mice were allowed to recover 14 days, followed by BrdU IP injections (50 mg/kg bodyweight) 1X daily for 5 days. One day after receiving the fifth BrdU IP injection, mice were saline and 4% PFA perfused. Immunostaining and Quantification described elsewhere.

Aged (18 month) C57BL6/J male mice received lateral intrahippocampal injections of 1 μl of lentiviral solutions (LV-shRNA-Smad3-GFP or LV-shRNA-lacZ-GFP, 1–3 × 10^8 IU/mL) in PBS on day −14, on the right hemisphere hippocampus, at 0.25 uL per minute. The injection coordinates with respect to bregma were −2.12 anteriorposterior, −1.55 dorsoventral (from the dura), and 1.5 mediolateral (refer to Supplemental Figure 4). Mice were allowed to recover 14 days, followed by EdU IP injections (50 mg/kg bodyweight) 1X daily for 5 days. Five days after receiving the fifth EdU IP injection, mice were saline and 4% PFA perfused. Immunostaining and Quantification described elsewhere.

### shRNA muscle injection

Aged (24 month) C57BL6/J male mice were injured with CTX in the tibialis anterior (TA) muscle in two sites as described below, and on the following day 5 μl of concentrated lentiviruses carrying *Smad3* shRNA or *LacZ* shRNA were injected to the injury sites using a 30 gauge needle. Mice TAs were harvested 5 days post injury, as described below.

### *In vitro* validation of dnTGFBR2 vector

Neural progenitor cells (NPCs) were transduced with retroviruses carrying dominant negative TGFBR2 (Addgene, Cambridge, MA, http://www.addgene.org/12640/) which has a cytoplasmic truncation and a FLAG tag, or GFP control and cultured for 72 hours. GFP and dnTGFBR2-transduced cell lysates were prepared as described in western blotting methods, and probed on western blots for the FLAG-tagged dnTGFBR2 using anti-FLAG antibody (Santa Cruz), GFP (Abcam), pSmad3 (Epitomics), Smad2/3 (Santa Cruz), and βactin (Cell Signaling). Transduced NPCs were also plated at 300,000 cells per well of a 6-well tissue culture plate and cultured in basal medium (DMF12 + N2) after cell splitting for 4 hours, followed by 45 minute treatment with 0, 1, or 12.5 ng/mL TGF-β1. Cell pellets were lysed in 100 uL RIPA buffer, and probed on western blots for pSmad3, Smad2/3, and βactin.

For proliferation assay, NPCs were transduced with 3 μL concentrated retroviruses per 3 × 10^5^ cells, delivering dnTGFBR2 or GFP and cultured for one week. Transduced NPCs were then split at 80,000 cells per well into 8-well chamber slides and cultured overnight in growth medium (DMF12 + N2 + 10 ng/mL FGF-2) in the presence/absence of 10 ng/mL TGF-β1. 16 hours post addition of TGF-β1, cells were pulsed for 4 hours with EdU (30 μM). Cells were fixed with 4% PFA for 20 minutes, and immunostained for FLAG (Abcam), GFP (Abcam), EdU (Invitrogen), and Sox2 (Santa Cruz), as described in immunocytochemistry methods.

### *In vivo* muscle injection of dnTGFBR2 retrovirus

Aged (18 month) C57BL6/J male mice were injured with CTX in the tibialis anterior muscle in two sites and in the gastrocnemius muscle in four sites as described below, and 5 μl of concentrated retroviruses carrying dnTGFBR2 or GFP control were injected to the injury sites using a 30 gauge needle at different time points (see Figure 6 for details). Mice received EdU IP injection (50 mg/kg) 12 hours before muscle harvest at 5 days post injury.

### Muscle injury

Isoflurane was used to anesthetize the animal during the muscle injury procedure. For bulk myofiber satellite cell activation, gastrocnemius muscles were injected with cardiotoxin 1 (Sigma) dissolved at 100 micrograms per milliliter in PBS, at 4 sites of 5 microliters each for each muscle. Muscles were harvested 3 days later. For focal injury to assay regeneration by immunoanalysis and histology, 5 microliters of 0.5 milligram per milliliter CTX was injected to two sites at the middle of the tibialis anterior muscle, and muscle harvested 5 days later.

### Muscle fibers and muscle progenitor cell isolation

Injured gastrocnemius muscle was dissected from old mice and incubated at 37°C in digestion medium (150 U/mL Collagenase type II in DMEM medium, buffered with 30 mM HEPES) for 2 hours. Digested muscle was gently triturated and myofibers were collected. Myofibers were further digested with 1 U/mL Dispase and 40 U/mL Collagenase type II to liberate muscle stem cells [65]. Muscle stem cells were cultured in DMEM (Life Sciences) with 5% serum from the same age mouse.

### Muscle tissue immunofluorescence and histological analysis

Muscle tissue was dissected, flash frozen in OCT compound (Tissue Tek; Sakura) and cryo-sectioned at 10 micrometers. Cryo-sectioning was performed through the entire volume of muscle (50–70 sections total, done at 200 μm intervals), thereby serially sampling the entire tissue. Muscle sections were stained with aqueous hematoxylin and eosin (H&E), as per the manufacturer's instructions (Sigma-Aldrich). Regeneration and myogenic potential was quantified by examining injury sites from representative sections along the muscle spanning the injury, then by measuring the injured area using Adobe Photoshop Elements. Myofiber regeneration was quantified by counting total newly regenerated fibers and dividing by the regeneration area. Immunostaining was performed as described [69]. Briefly, after permeabilization in PBS + 1% FBS + 0.25% Triton-X-100, tissues and cells were incubated with primary antibodies in staining buffer (PBS + 1% FBS) for 1 h at room temperature, followed by 1 h incubation fluorochrome-labeled secondary antibodies (ALEXA at 1:1000). BrdU-specific immunostaining required an extra step of 2 M HCl treatment before permeablization.

### Whole muscle tissue analysis

Whole muscle from young and old resting TA and TA adjacent to cardiotoxin injured gastrocnemius muscle was isolated and lysed as described earlier [7]. In brief, muscles were lysed using Miltenyi Biotec Tissue Dissociator in tissue lysate buffer and lysates were run on 7.5% Criterion gel (BioRad) and western blotted against TGF-β1 (R&D, mouse monoclonal antibody) and GAPDH (Abcam, goat polyclonal) antibody. The images were quantified using Image J software and P values calculated using student's *t*-test.

### Quantification and statistical analysis

For quantification of immunofluorescent images for BrdU incorporation, 25 20x images per replicate were taken on the Molecular Devices ImageXpress Micro automated epifluorescence imager, followed by automated cell quantification using the multiwavelength cell scoring module within the MetaXpress analysis software. Data was analyzed using Student's *t*-tests (two-tailed) and P values equal or lower than 0.05 were considered statistically significant. Sample sizes of *n* = 3 or greater were determined for each experiment based on power analysis and IACUC considerations, and based on previously published experimental group numbers [19, 44, 63] and assessed for significance based on p values and heteroscedastic variance between groups that were statistically compared. For pixel intensity and percent area quantifications of immunofluorescent images, MetaXpress Analysis Software or ImageJ were used to determine integrated pixel intensity and percent positive area of thresholded images.

For quantification of the number of BrdU+Sox2+ cells in Alk5 inhibitor treated mice, unbiased stereology (Zeiss Axio Imager, software by MicroBrightfield) using the optical fractionator method was performed on 8 vibratome coronal brain slices spanning the hippocampus (40 microns thick, 200 microns apart), and the number of selected cells was normalized by the volume of hippocampal tissue analyzed. For quantification of the number of BrdU+Sox2+ or EdU+DCX+ cells in shRNA viral vector injected brains, confocal stacks of 8 vibratome coronal GFP+ brain slices spanning the hippocampus (40 microns thick, 200 microns apart) were acquired on a Prairie confocal microscope and cells were counted. Cell numbers were normalized to the volume of the DG granule cell layer measured by ImageJ and as previously described [59]. Briefly, volume was calculated based on a threshold of the granule layer of each image as determined with Hoechst staining, then calculating the volumetric fraction based on the thickness of the brain slice (40 μM) and the interval at which hippocampi sections were analyzed (every 6^th^ section).

### Inclusion/exclusion criteria

Only aged mice that died during the study due to health reasons were excluded from analysis. In terms of automated counting of cells using MetaXpress analysis software, only sites that were blurry with indistinguishable colors (such as areas on the slide with bubbles or sites imaged with incorrect focus by the Molecular Devices ImageXpress Micro automated epifluorescence imager), or areas with large cell clumps were excluded from the cell quantification analysis. These criteria were pre-established.

When performing *in vivo* experiments, such as Alk5 inhibitor injections or shRNA steretaxic brain injections, there was no blinding. For quantification and analysis, researchers were blinded to the group allocation, specifically when doing regenerative index calculations, stereology and cell counts.

## SUPPLEMENTARY FIGURES AND TABLES


