# Supplementary material for: Systemic attenuation of the TGF-β pathway by a single drug simultaneously rejuvenates hippocampal neurogenesis and myogenesis in the same old mammal
Source: Oncotarget. 2015 May 6;6(14):11959–78. doi: 10.18632/oncotarget.3851 (PMC4494916; doi:10.18632/oncotarget.3851)
Supplement: Supplementary file 1 [file oncotarget-06-11959-s001.pdf]

SUPPLEMENTARY FIGURES AND TABLE

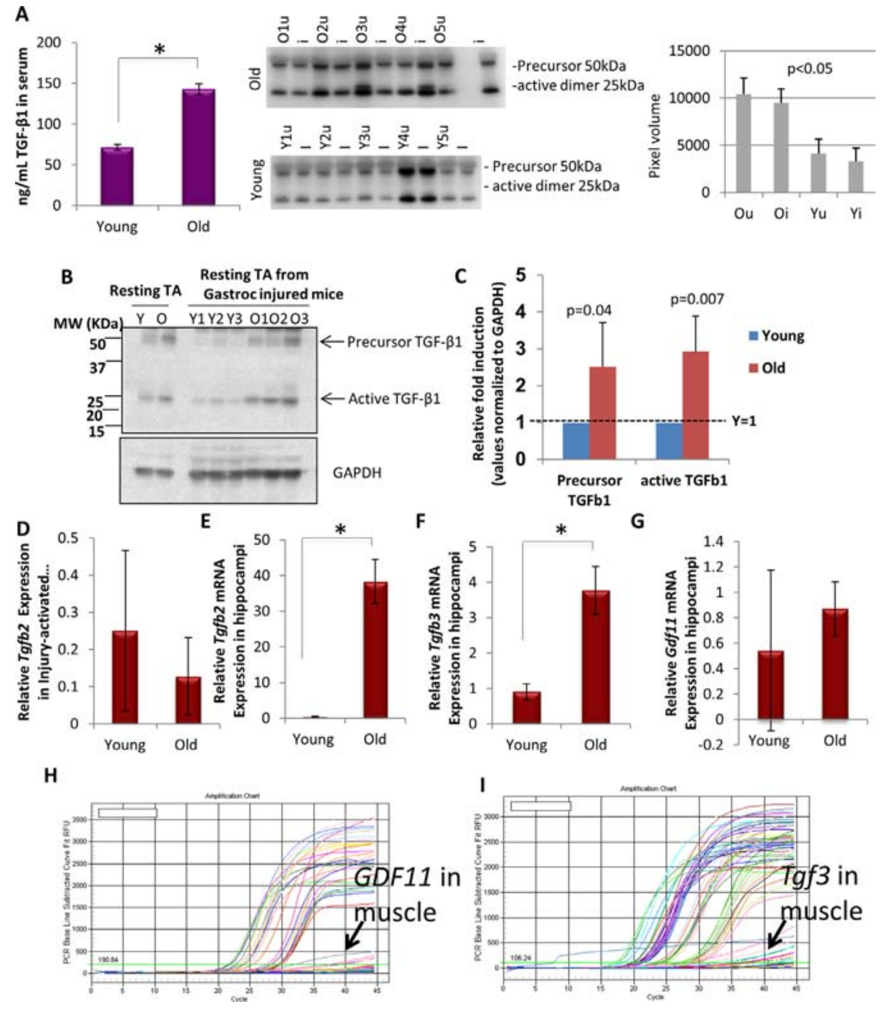

Supplementary Figure S1: TGF-β Levels are Increased Systemically in Serum, and in Aged Muscle and Hippocampi. A. An ELISA was performed on young and old serum to assess the average level of systemic TGF-β1, represented as pg/mL. Significant differences were identified by Student's *t*-tests (two-tailed) (*$p < 0.003$). Error bars indicate standard error of the mean ($n = 3$ biological replicates per group). One microliter of serum samples from young (Y) and old (O) mice, that were injured (i) or uninjured (u), were also analyzed by western blotting for TGF-β1; the ECL images were quantified by ImageJ and plotted as mean background-corrected pixel volume + 1 S.E.M, $p < 0.05$ between young and old, $n = 5$. **B.** Whole muscle from young and old resting TA and resting TA adjacent to cardiotoxin injured gastrocnemius muscle was isolated and western blotting against TGF-β1 antibody was performed on muscle samples. GAPDH served as control. No differences were observed between the levels of TGF-β1 in the TA from completely non-injured leg and those levels in non-injured TA adjacent to injured gastrocnemius. As shown, aged muscle had significantly more TGF-β1 as compared to young. **C.** TGF-β1 quantification graph. TGF-β1 levels from panel A were quantified and relative fold induction after GAPDH normalization was plotted as a histogram (Youn $g = 1$) ($n = 4$ biological replicates, *$p \leq 0.05$). **D.** qRT-PCR quantification of *Tgfb2* mRNA expression from young and old injury-activated myofibers. The relative average expression level was normalized to *GAPDH* and presented as the average expression level relative to that of young myofibers. There were no differences in expression level with age. **E.** qRT-PCR quantification of *Tgfb2* mRNA expression from young and old hippocampi. The relative average expression level was normalized to *GAPDH* and presented as the average expression level relative to that of young hippocampi. Significant differences were identified by Student's *t*-tests (two-tailed) (*$p < 0.04$). Error bars indicate standard error of the mean ($n = 4$ biological replicates per group). **F.** qRT-PCR quantification of *Tgfb3* mRNA expression from young and old hippocampi. The relative average expression level was normalized to *GAPDH* and presented as the average expression level relative to that of young hippocampi. Significant differences were identified by Student's *t*-tests (two-tailed) (*$p < 0.03$). Error bars indicate standard error of the mean ($n = 4$ biological replicates per group). **G.** qRT-PCR quantification of *Gdf11* mRNA expression from young and old hippocampi. The relative average expression level was normalized to *GAPDH* and presented as the average expression level relative to that of young hippocampi. There were no differences in expression level with age. **H.** Amplification chart of *Gdf11* qPCR in injury-activated myofibers demonstrates no amplification, as demonstrated by lack of CT values for *Gdf11* **I.** Amplification chart of *Tgfb3* qPCR in injury-activated myofibers demonstrates no amplification, as demonstrated by lack of CT values for *Tgfb3*.

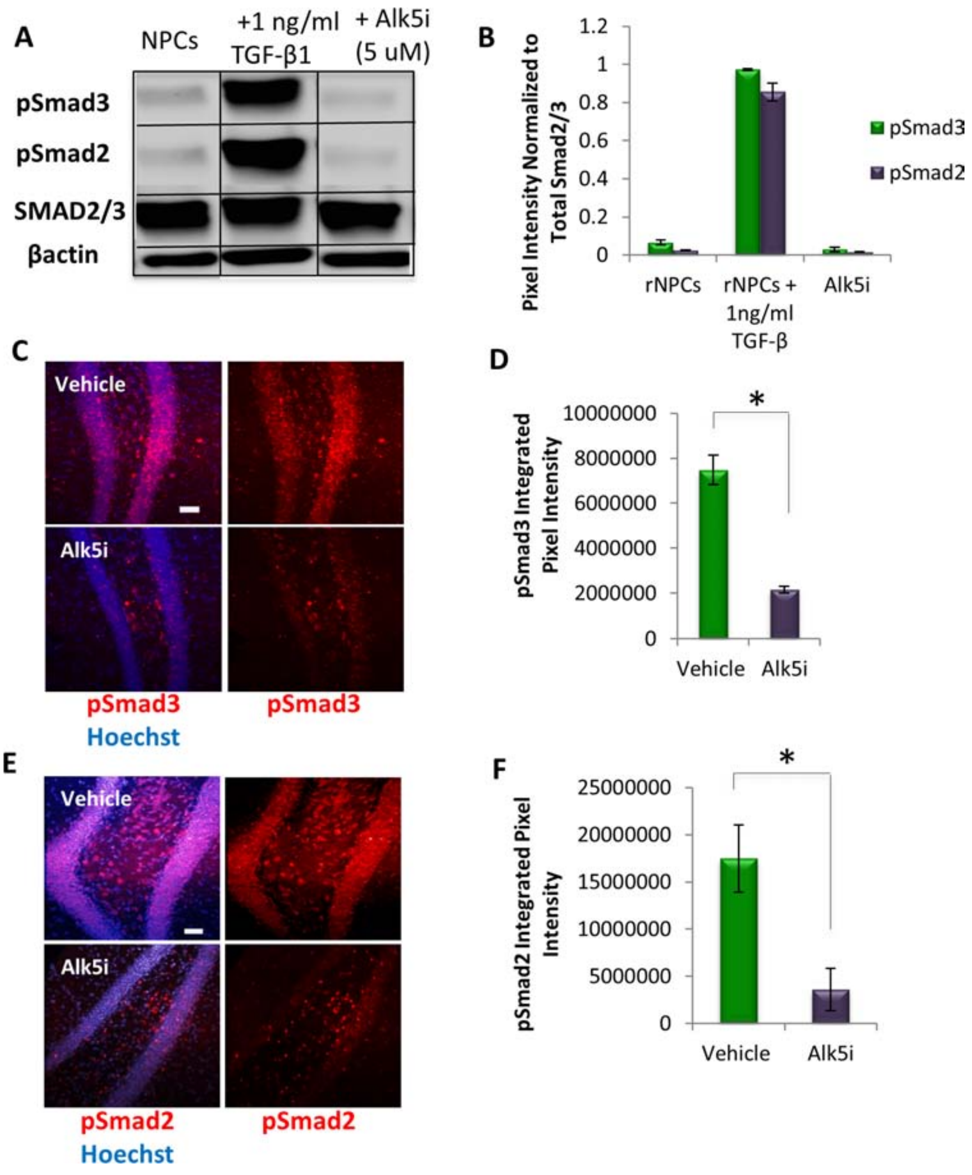

Supplementary Figure S2: Alk5 Inhibitor Reduces the Levels of pSmad2/3 in Primary Neural Progenitor Cells *in vitro* and in the Murine Dentate Gyrus after Systemic Administration *in vivo*. A. NPCs were incubated in basal medium (DMF12 + N2 but no FGF-2) for 1 hour, then either untreated, or treated with TGF-β1 (1 ng/mL) for 1 hour +/- 5 uM Alk5 inhibitor. Representative western blotting for pSmad2 and 3 is shown. Note that lanes have been cropped for presentation purposes, indicated by the lines separating the lanes. **B.** Quantification of immunoblot from (A), represented as the mean pixel intensity of pSmad2 or pSmad3 normalized to total SMAD2/3 (n = 2 biological replicates). **C.** Following systemic delivery of Alk5 inhibitor or control vehicle, immunofluorescence was performed on brain sections spanning the hippocampus for pSmad3 (red), with hoechst (blue) labeling cell nuclei. Representative images are shown. Scale bar = 50 μM. **D.** pSmad3 integrated fluorescence intensity was quantified in brain sections using MetaXpress software. Significant differences were identified by Student's t-tests (two-tailed) (*$p < 0.05$). Error bars indicate standard error of the mean (n = 4 biological replicates (mice), with n = 6 technical replicates per mouse). **E.** pSmad2 (red) immunofluorescence was performed on brain sections spanning the hippocampus following systemic delivery of Alk5 inhibitor or control, with hoechst (blue) labeling cell nuclei. Representative images are shown. Scale bar = 50 μM. **F.** pSmad2 integrated fluorescence intensity was quantified in brain sections using MetaXpress software. Significant differences were identified by Student's t-tests (two-tailed) (*$p < 0.05$). Error bars indicate standard error of the mean (n = 4 biological replicates (mice), with n = 6 technical replicates per mouse).

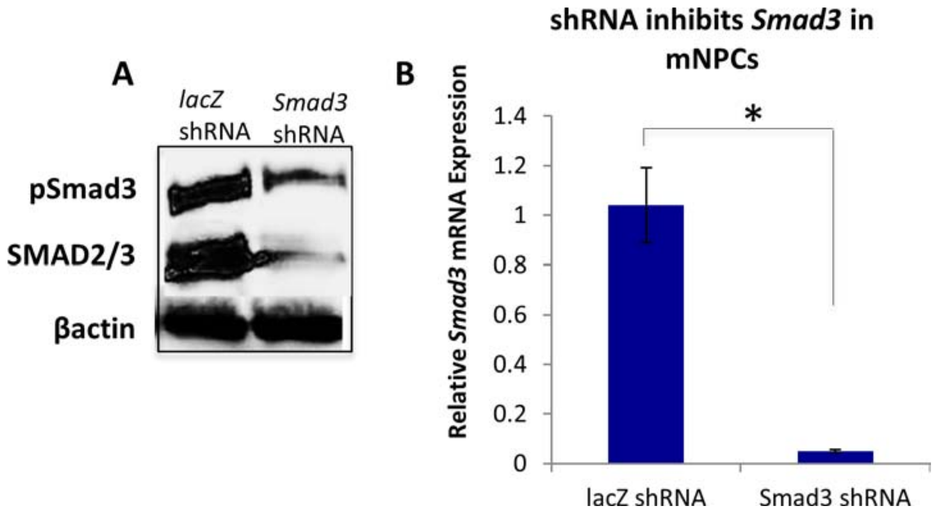

Supplementary Figure S3: *In vitro* Validation of *Smad3* shRNA. A. Mouse muscle progenitor cells were transduced with 1 μl of concentrated *Smad3* shRNA or *lacZ* shRNA lentiviruses and cultured for 72 hours, followed by western blot analysis of pSmad3 expression and total SMAD2/3. *Smad3* shRNA inhibited expression of SMAD3. βactin was used as a loading control. **B.** Quantification of *Smad3* mRNA expression by qRT-PCR was performed on RNA extracted from mouse NPCs transduced with shRNA to *Smad3* or control virus and passaged for 2 weeks. The relative expression level was normalized to *GAPDH* and presented as the expression level relative to that of NPCs transduced with *lacZ* shRNA lentivirus. Significant differences were identified by Student's *t*-tests (two-tailed) (*$p < 0.04$). Error bars indicate standard error of the mean ($n = 3$ biological replicates).

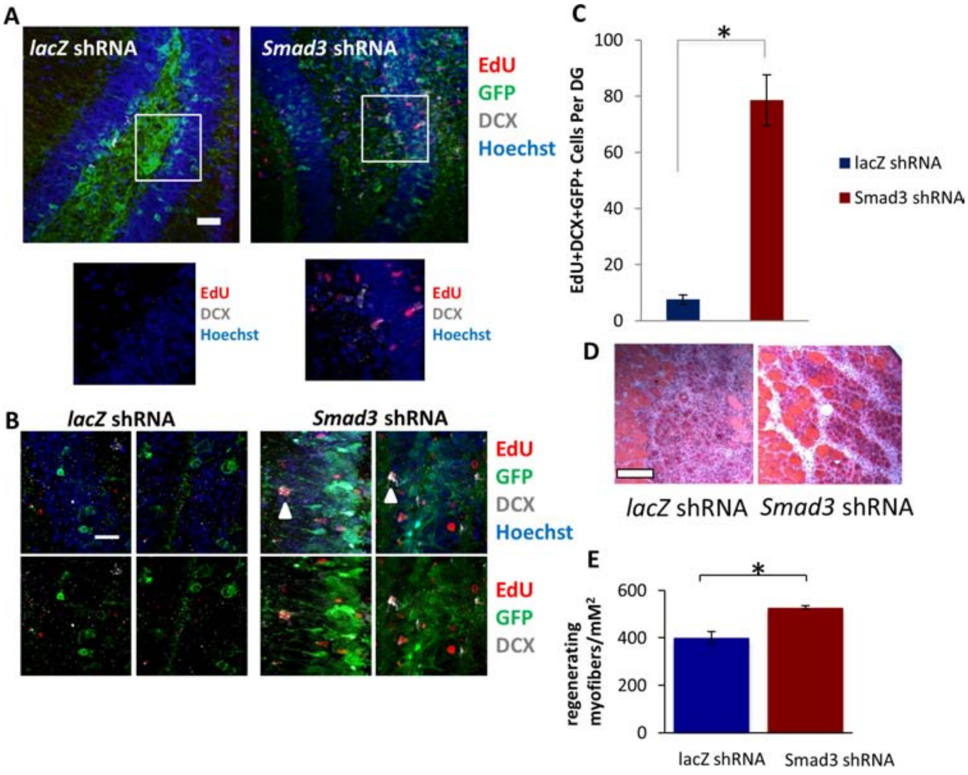

Supplementary Figure S4: *Smad3* Inhibition via *Smad3* shRNA Enhances Neurogenesis and Myogenesis of Old Tissue. A. Aged (18 month old) mice received stereotaxic injections into hippocampi (coordinates from bregma: AP: −2.12, ML: +/−1.5, VD: −1.55) of lentiviral vectors encoding either shRNA against *Smad3* or shRNA against *lacZ*. The mice were allowed to recover for 14 days, followed by daily EdU (50 mg/kg) intraperitonial injections for 5 days. Five days after the last EdU injection, mice were saline and 4% PFA perfused. Brain sections of *lacZ* or *Smad3* shRNA injected mice ($n = 3$ *lacZ* shRNA, 5 *Smad3* shRNA) spanning the entire hippocampus were immunostained with GFP (green), EdU (red), and Doublecortin (DCX) (gray), with Hoechst (blue) labeling cell nuclei. Representative images are shown. Scale bar = 50 µM. **B.** High magnification representative images as described in (A). Arrows indicate proliferating neuronaly committed precursor cells. Scale bar = 100 µM. **C.** Quantification of the mean number of EdU+DCX+GFP+ cells per dentate gyrus shows an increase in proliferating cells in *Smad3* shRNA injected aged murine hippocampi. Significant differences were identified by Student's *t*-tests (two-tailed) ($*p < 0.03$). Error bars indicate standard error of the mean ($n = 3$ *lacZ* shRNA, 5 *Smad3* shRNA). **D.** Representative hematoxylin and eosin stained 10-µm cross-sections of tibialis anterior muscle 5 days after cardiotoxin induced injury in the presence of *Smad3* shRNA or control *lacZ* shRNA lentiviral particles. **E.** Quantitative analysis of the number of newly formed fibers normalized to cross-sectional area of regenerating tissue in control transduced and *Smad3* shRNA-transduced tibialis anterior muscle 5 days after injury. Centrally nucleated myofibers were scored from hematoxylin and eosin stained cryosections (10 µM sections throughout the site of injury) ($n = 3$ mice per group, with $n = 3$ technical replicates per mouse, mean ± SEM) ($*p < 0.0001$).

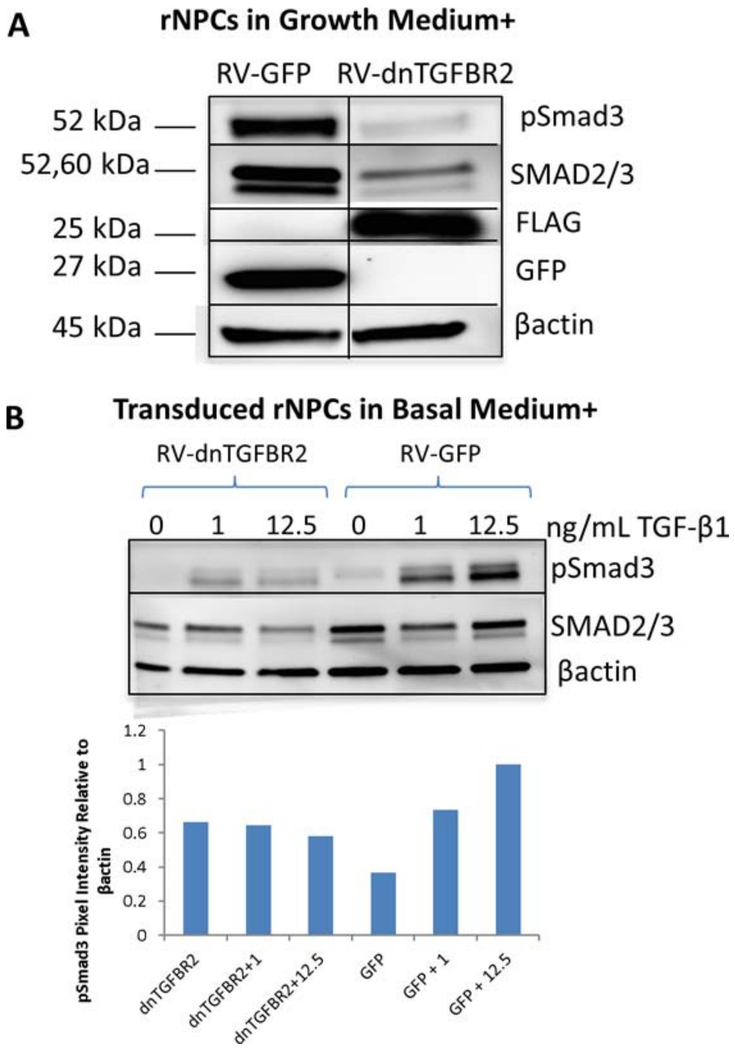

Supplementary Figure S5: Expression of dnTGFBR2 in Neural Progenitor Cells. A. Neural progenitor cells were transduced with retrovirus delivering dnTGFBR2-FLAG or GFP. After 72 hours in growth (FGF-2 rich) medium, cells were lysed. Western blotting was performed with an anti-FLAG antibody specific for the C-terminal fusion of the dnTGFBR2, GFP, pSmad3, SMAD2/3, and βactin as a loading control. As shown, the 22 kDa FLAG/dnTGFBR2 band was detected in RV-dnTGBR2 transduced cells, and GFP was detected in RV-GFP transduced neural progenitor cells. dnTGFBR2 expression resulted in downregulation of pSmad3, as well as total SMAD2/3 after 72 hours. Note that lanes have been cropped for presentation purposes, indicated by the line separating the lanes. **B.** Neural progenitor cells were cultured for one week post retroviral transduction, then incubated for four hours in basal (DMF12+N2, no FGF-2) medium after splitting. Cells were then either treated or not with 1 or 12.5 ng/mL TGF-β1 for 45 minutes. pSmad3, SMAD2/3, and βactin were assessed via western blot. pSmad3 pixel intensity relative to βactin was quantified with Bio-Rad imaging software.

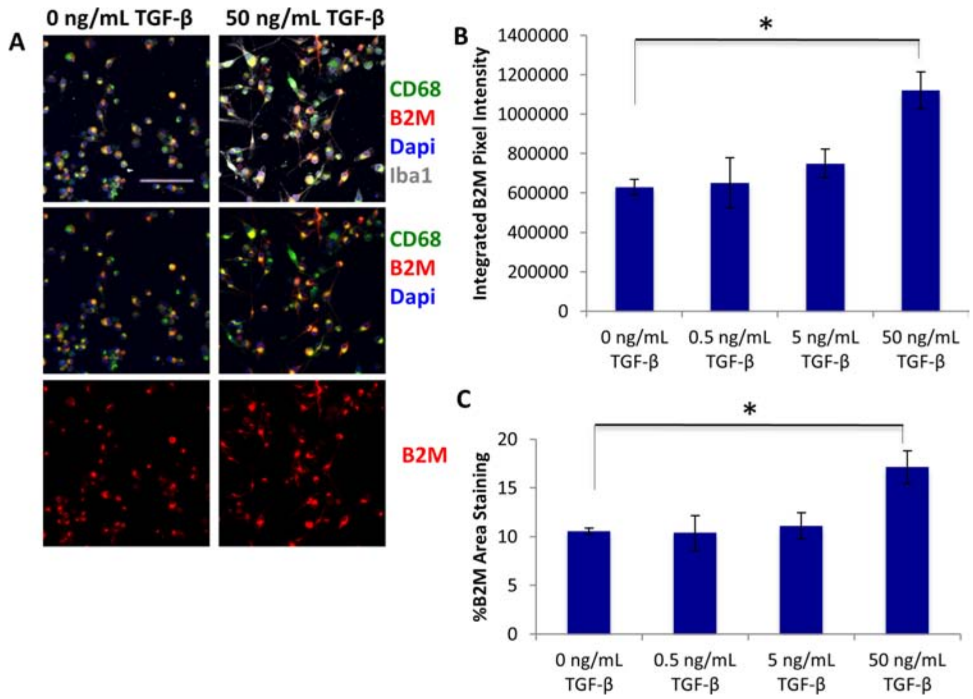

Supplementary Figure S6: High concentration of TGF-β upregulates B2M expression in immune cells. A. BV2 cells were cultured in basal medium (DEMEM) and treated for 24 hours with LPS (100 ng/mL) and IFN-03D2 (20 ng/mL) +/- varying concentrations of TGF-β (0, 0.5, 5, and 50 ng/mL), followed by PFA fixation and immunohistochemistry analysis. Immunofluorescence (IF) was performed for microglial markers CD68 (green) and Iba1 (gray), as well as MHCI molecule B2M (red), with DAPI (blue) labeling all nuclei. Representative images are shown. Scale bar = 100 µM. **B.** Integrated pixel intensity of B2M immunofluorescence in treated BV2 cells was calculated using ImageJ. Significant differences were identified by Student's *t*-tests (*$p < 0.05$). Error bars indicate standard error of the mean ($n = 4$). **C.** Percent B2M area staining was calculated using ImageJ. Significant differences were identified by Student's *t*-tests (*$p < 0.05$). Error bars indicate standard error of the mean ($n = 4$).

Supplementary Table S1. shRNA Sequences

	shRNA and qPCR Primer Sequences
shRNA Smad 3.1	5′-GCCCATGTTTCTGCATGGATTTCTCTTCAAGAGAGAGAAATCCATGCAGAAACATGGGCTTTTT-3′
shRNA Smad 3.2	5′-GCTGTCCAATGTCAACCGGAATCTCTTCAAGAGAGAGATTCCGGTTGACATTGGACAGCTTTTT-3′
shRNA Smad 3.3	5′- GCATCCGTATGAGCTTCGTCAACTCTTCAAGAGAGAGTTGACGAAGCTCATACGGATGCTTTTT-3′
shRNA Smad 3.4	5′-GGACCTGAGTGAAGATGGAGATTCAAGAGATCTCCATCTTCACTCAGGTCTTTTT-3′
shRNA Smad 3.5	5′-GGGTCCAATGTCAACCGGAATTTCAAGAGAATTCCGGTTGACATTGGACCTTTTT-3′
shRNA lacZ	Sense: 5′-GGGGTTAATTAAAAGGTCGGGCAGGAAGAGGGC-3′<br>Antisense: 5′-GGGGTTAATTAAAAAAAGTGACCAGCGAATACCTGTTCTC-3′
Tgfb1	F: 5′-GCT ACC ATG CCA ACT TCT GT -3′; R: 5′-CGT AGT AGA CGA TGG GCA GT-3′
p21	F: 5′-CCG CGG TGT CAG AGT CTA-3′; R: 5′-CAT GAG CGC ATC GCA ATC-3′
Smad3	F: 5′-ACC AAG TGC ATT ACC ATCC-3′; R: 5′-CAG TAG ATA ACG TGA GGG AGC CC-3′
Tgfb2	F: 5′-CCT GAC CGC TCT GAG AAT TA-3′ ; R: 5′-ATC CTC TTG CGC ATA AAC TG -3′
Tgfb3	F: 5′-AGG GTG TAT TCT CCG GAT TC-3′ ; R: 5′-ATC CAT GAT TCC CCA AAA AT -3′
Gdf11	F: 5′-CCC CAA TCA ACA TGC TCT AC-3′; R: 5′-TGA TGT TCA TGG GAA GAG GT -3′
B2M	F: 5′-GGC CTG TAT GCT ATC CAG AA -3′; R: 5′- GAA AGA CCA GTC CTT TGC TGA -3′
GAPDH	F: 5′-CTGGAGAAACCTGCCAAGTA-3′ R: 5′-TGTTGCTGTAGCCGTATTCA-3′
